# Risk of Anticoagulation in Gluteal Compartment Syndrome After Surgical Intervention

**DOI:** 10.7759/cureus.50233

**Published:** 2023-12-09

**Authors:** José R Oliveira, João Dinis, André Sarmento, David Sá, Rui Lemos

**Affiliations:** 1 Orthopedics and Traumatology, Centro Hospitalar de Vila Nova de Gaia/Espinho, Vila Nova de Gaia, PRT

**Keywords:** femur intertrochanteric fracture, pelvic trauma, perioperative complications, fasciotomy, sciatic neuropathy, hypocoagulation, acute compartment syndrome, gluteal compartment syndrome

## Abstract

Gluteal compartment syndrome (GCS) is a rare form of acute compartment syndrome. There are some causes, such as prolonged periods of immobilization and traumatic or iatrogenic events. We report two cases of gluteal compartment syndrome after orthopedic surgical intervention for fracture stabilization. The patients were both hypocoagulated due to the presence of two mechanical heart valves. Despite early treatment, both patients remained with neurological deficits. Orthopedic and trauma surgeons must be aware of the possibility of gluteal compartment syndrome in perioperative patients. Recognizing and managing risk factors such as hypocoagulation is crucial for its prevention.

## Introduction

Acute compartment syndrome is a condition that develops when increased pressure within a myofascial compartment surpasses the capillary perfusion pressure, inducing ischemic lesion to muscles. Muscle edema can compress surrounding structures, including peripheral nerves, resulting in compressive mononeuropathies [1-5]. Gluteal compartment syndrome (GCS) was first described in 1977 by Evanski et al. and represents a limb and potentially life-threatening emergency. It is a rare type of compartment syndrome; therefore, a high index of suspicion is required for prompt diagnosis and management [6,7]. The causes of this condition include prolonged immobilization, trauma, or iatrogenic injury [8].

The delay in diagnosis has been shown to lead to complications, including residual motor or sensory deficits [8]. An established treatment protocol has yet to be determined. Decompressive fasciotomy is frequently regarded as the therapy of choice for gluteal compartment syndrome; however, there have been a limited number of cases in which patients have improved through the implementation of conservative measures [2,4]. In this report, we describe two cases of GCS in two hypocoagulated patients after an orthopedic intervention.

## Case presentation

Approval by an ethics committee or institutional review board was not necessary because this was a non-invasive follow-up observational study. We obtained verbal informed consent from all patients in the study. 

Case 1

A 55-year-old male patient was brought to another institution after a motor vehicle accident. As per the history received, the patient had a mechanical aortic valve due to rheumatoid aortic valve disease, receiving anticoagulant treatment with warfarin and antiplatelet therapy with aspirin. The patient was presented with a complaint of intense pain in the right hip. The advanced trauma life support (ATLS) protocols were followed. Physical examination revealed an unstable pelvis, deformity, and pain in the right thigh. A pelvic binder was used to stabilize the pelvis. Pelvic and thigh radiographs revealed a comminuted fracture of the left sacral ala, left superior and inferior pubic ramus, and a right femoral shaft fracture. Computer tomography (CT) showed no intracranial or cervical spine injury and confirmed the pelvic fractures: a left superior pubic ramus with involvement of the anterior acetabular wall, an inferior pubic ramus without displacement, and a comminuted left sacral ala fracture. There were no signs of bleeding within the pelvis. The hemoglobin level on admission was 13.2 g/dl. A pelvic external fixation and a retrograde nailing of the right femur were performed in the context of damage control.

**Figure 1:**
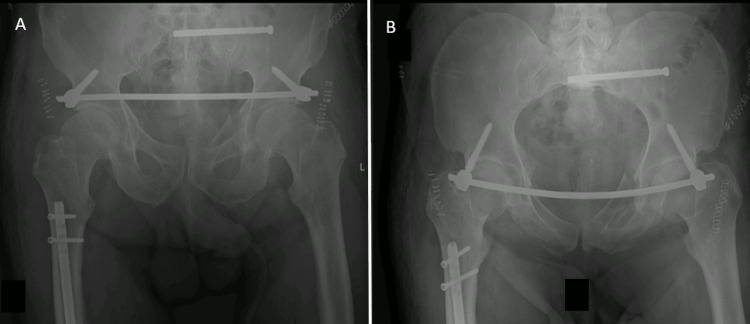
Anteroposterior (A) and inlet (B) radiographs of the pelvis showing the correct position of the INFIX and iliosacral screw INFIX - anterior pelvic internal fixator

Three days after the initial stabilization, the patient was transferred to our institution. He was hemodynamically stable, and the hemoglobin level was unchanged. He was anticoagulated with unfractionated heparin with an international normalized ratio (INR) of 1.7. A percutaneous approach to the pelvis was preferred due to the risk of bleeding. A subcutaneous anterior fixator (INFIX - anterior pelvic internal fixator) and a left iliosacral screw was placed (Figure 1).

**Figure 2:**
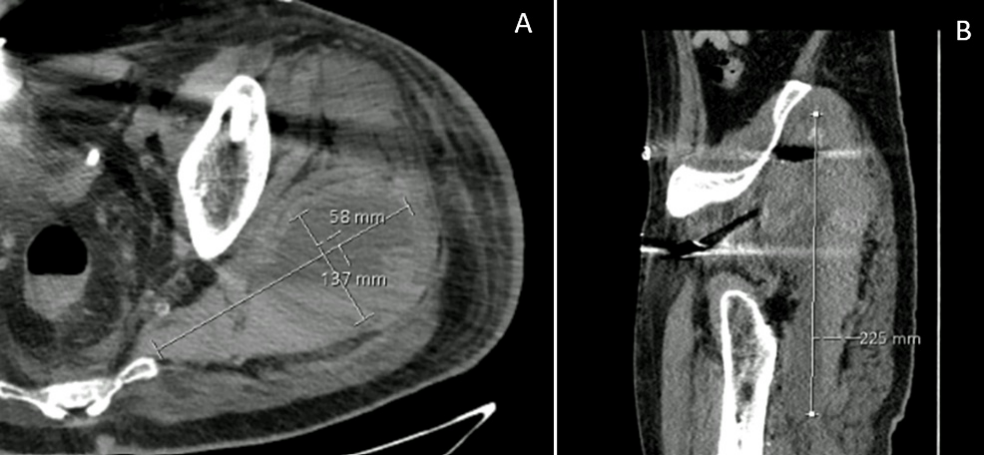
Axial (A) and sagittal (B) CT scans showing a large hematoma in the left gluteal region

Unfractionated heparin was resumed after 24 hours of intervention at a therapeutic dosage because of the thromboembolic risk. On the fourth day after the surgery, he started to complain of pain and swelling in the left buttock region. A CT scan was required at this stage. The symptoms progressively worsened, and after six hours, he was in excruciating pain, unable to move in bed. At the physical examination, he presented with extreme pain with passive hip motion, a swollen tense left buttock, lost the left ankle flexion and extension, and the sensibility of the lateral portion of the leg and foot, suggesting sciatic nerve palsy. The computed tomography revealed a large hematoma (225x137x58 mm) in the left gluteal region without signs of arterial bleeding (Figure 2).

The patient was taken emergently to the operating room. A Kocher-Langenbeck approach in a lateral decubitus position was performed [9-11]. An extensive hematoma was encountered deeper into the gluteus maximus and promptly removed. The hemostasis was achieved, and no important bleeding vessels were found. The sciatic nerve was identified and protected during the entire surgery. Its appearance indicated some damage. A drain was placed, and the incision was closed primarily. The unfractionated heparin was suspended for seven days to improve the bleeding control in the surgical area.

Post-surgery, the patient remained hemodynamically stable with negligible alteration in his hemoglobin levels. The sensory deficits resolved within the first 72 hours. Physical therapy was started promptly. Nevertheless, after 18 months, the patient remained with a sciatic motor deficit with a persistent foot drop (1/5 strength with testing of the anterior tibialis according to the Medical Research Council scale) [12].

Case 2

**Figure 3:**
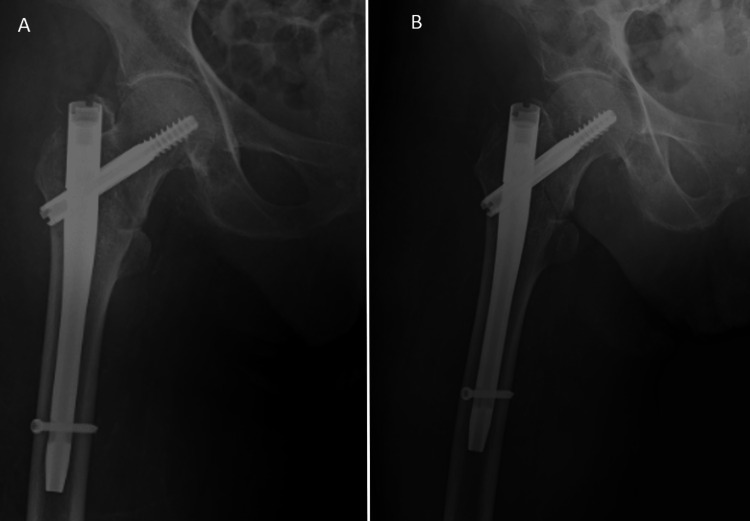
Postoperative anteroposterior (A) and oblique (B) radiographs of the right hip

An 84-year-old female was admitted to our emergency department after a fall from height. She was complaining of pain in the right hip and was unable to walk. The physical examination revealed a shorter and externally rotated right lower limb. The radiograph evaluation revealed a right intertrochanteric fracture. As part of her medical history, she suffered from valvular heart disease and had a mechanical aortic valve, receiving acenocoumarol to prevent blood clotting. The INR was 2.2. After two days of acenocoumarol suspension, the INR normalized, and the patient was taken to the operating room. Under C-arm fluoroscopy, the intertrochanteric fracture was accurately reduced by closed means and was fixed with a 125° cephalomedullary nail (Figure 3).

**Figure 4:**
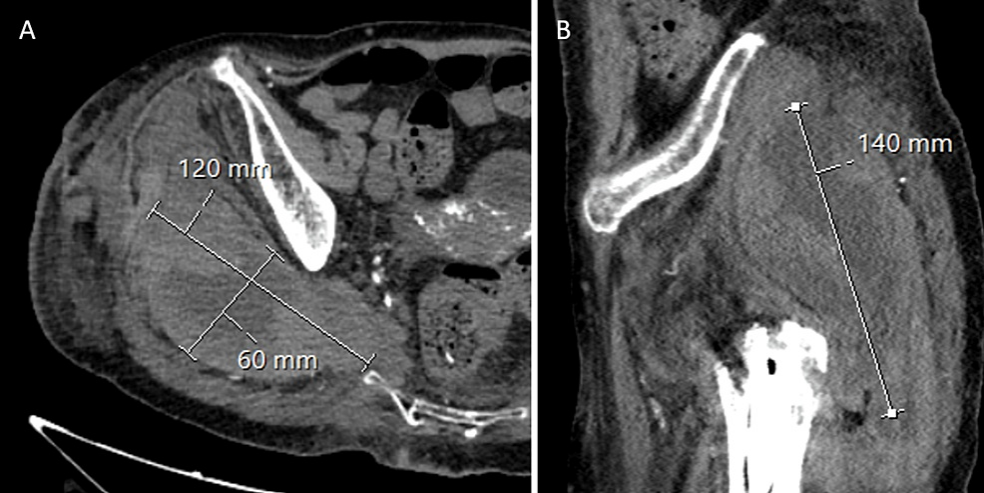
Axial (A) and sagittal (B) CT scans showing a large hematoma in the right gluteal region

Anticoagulation treatment with enoxaparin at a therapeutic dosage was resumed 24 hours after the procedure. The rehabilitation protocol, which included immediate walking with crutches for partial weight-bearing, started 48 hours after the surgery. On the third postoperative day, the patient began to express discomfort of a moderate degree in the region of the right buttock. A request was made for a CT scan and blood tests. The hemoglobin level dropped from 9 to 6.3 g/dl, and the CT scan showed an extensive hematoma (120x140x60 mm) without any signs of active arterial bleeding (Figure 4). After 12 hours, the patient no longer felt the lateral aspect of her lower limb and foot, in addition to an inability to move her ankle, findings that were suggestive of sciatic palsy.

Urgent fasciotomy was performed to decompress the gluteal compartment. A Kocher-Langenbeck approach was used [9-11]. A large hematoma was drained. Careful hemostasis was performed, yet no discernible origin of active hemorrhage was identified. The sciatic nerve exhibited visual characteristics suggestive of certain impairment. The incision was closed, and a vacuum dressing was used to aid wound closure. The patient remained without anticoagulation treatment for seven days.

Following a 12-month period of observation, the patient did not exhibit any impairments in sensory function while still retaining a notable impairment in the ability to dorsiflex the ankle (1/5 strength).

## Discussion

The occurrence of gluteal compartment syndrome is infrequent and has the potential to result in severe consequences for the patient. The anatomy of the gluteal region was well described by David et al. and contains three non-expandable compartments: gluteus maximus compartment, gluteus medius and minimus compartment, and tensor fascia lata compartment [9,13].

The most common etiology is perioperative immobilization. In the postoperative course, these periods of restraint increase the pressure applied to the gluteal compartment, leading to ischemia, edema, and the subsequent development of acute compartment syndrome [14-17]. The perioperative features associated with the emergence of gluteal compartment syndrome encompass the length of the surgical procedure, patient body habitus, use of epidural anesthesia, and positioning during the operation [4]. Certain papers highlight hypocoagulation as a potential risk factor [18-20]. The two patients were hypocoagulated due to the presence of mechanical heart valves. Considering the potential for thromboembolic complications, the anticoagulation therapy was reintroduced at a therapeutic dosage within 24 hours after the procedure. The authors hold the conviction that the anticoagulation treatment constituted an important risk factor for the development of this condition. In the perioperative period, it is imperative to exercise caution while administering this medication by carefully considering the thromboembolic risks in relation to the potential for hemorrhage. A multidisciplinary discussion involving immunohemotherapy sepcialists, cardiologists, and anesthetists may prove to be beneficial in establishing the optimal regimen for the postoperative management of these patients.

Trauma is also one of the most common causes of GCS [1,13]. There are some described cases associated with pelvic fractures and also one case associated with hip dislocation [21-26]. We believe the second case presented here to be the first reported case of gluteal compartment syndrome as a result of intertrochanteric fracture.

GCS is an under-reported and under-diagnosed orthopaedic emergency. The symptoms include pain, edema, and ecchymosis in the gluteal region [8,27,28]. Sciatic palsy may subsequently develop, with associated motor and sensory deficits. Measurement of compartment pressures may be helpful. However, if there is clinical suspicion, prompt treatment is advocated, regardless of confirmatory pressure measurement [8,9,22,28-30]. These two cases demonstrate how quickly the condition evolves, with neurological deficits developing a few hours after the onset of symptoms. A high index of suspicion is necessary to achieve a timely diagnosis. 

The classic treatment for acute compartment syndrome is emergent fasciotomies. The release of the three compartments is vital in the treatment of this condition [31]. The surgical technique that is most frequently reported for decompression involves a modification of the Kocher-Langenbeck approach [9-11]. Some papers suggest that early treatment with fasciotomies improves the chances for full recovery [32,33]. Nevertheless, a recent systematic review suggests that there is no difference in rates of permanent neurological deficits between surgical and medical treatment in patients presenting without an initial neurological deficit. The authors propose a treatment algorithm where the neurological impairments are accounted for the necessity of conducting fasciotomies [4]. Unfortunately, in these two cases, permanent neurological deficit could not be avoided despite the early treatment.

## Conclusions

Orthopedic and trauma surgeons must be aware of the possibility of GCS in postoperative patients. It has a high mortality and morbidity rate and must be kept in mind in the differential diagnosis. A prompt diagnosis can provide an early treatment and thus prevent permanent functional damage. One should be aware of the risk factors associated with its appearance, which should include the use of anticoagulant medication. Proper management of these risk factors is imperative to prevent this event. Urgent fasciotomy appears to be the appropriate treatment, especially for patients who present with neurological deficits. Additional research is needed in order to diagnose and treat this condition properly. The development of guidelines for managing high-risk patients may help prevent GCS.
